# Inflammation and fibrosis in Crohn’s disease: location-matched histological correlation of small bowel ultrasound features

**DOI:** 10.1007/s00261-020-02603-6

**Published:** 2020-06-20

**Authors:** Gauraang Bhatnagar, Manuel Rodriguez-Justo, Antony Higginson, Paul Bassett, Alastair Windsor, Richard Cohen, Steve Halligan, Stuart A. Taylor

**Affiliations:** 1 Centre for Medical Imaging, University College London, 2nd Floor Charles Bell House, 43-45 Foley Street, London, W1W 7TS UK; 2 Department of Histology, University College Hospitals, London, UK; 3 Department of Radiology, Queen Alexandra Hospital, Portsmouth, UK; 4 Statsconsultancy Limited, Amersham, UK; 5 Department of Colorectal Surgery, University College Hospitals, London, UK

**Keywords:** Crohn’s disease, Ultrasound, Inflammation, Fibrosis

## Abstract

**Purpose:**

To evaluate the utility of mural and extramural sonographic features of Crohn’s Disease as potential imaging biomarkers of inflammation and fibrosis against whole-mount histological sections.

**Methods:**

Twelve Crohn’s disease patients (Mean age 35(25–69), 7 males) underwent small bowel ultrasound prior to small bowel resection. Two radiologists in consensus graded multiple parameters including mural, mucosal and submucosal thickness, submucosal/mesenteric echogenicity and clarity and mural Doppler signal in 50 selected bowel cross-sections. Matching with histological sampling sites was facilitated via scanning of the resected specimen. A histopathologist scored acute and chronic inflammation, and fibrosis (using histological scoring systems) following analysis of whole mount block sections. The association between sonographic observations and histopathological scores was examined via univariable and multivariable analysis.

**Results:**

In univariate analyses, bowel wall thickness (regression co-efficient and 95% CI 0.8 (0.3, 1.3) *p* = 0.001), mesenteric fat echogenicity (8.7(3.0, 14.5) *p* = 0.005), submucosal layer thickness (7.4(1.2, 13.5) *p* = 0.02), submucosal layer clarity (4.4(0.6, 8.2) *p* = 0.02) and mucosal layer thickness (4.6(1.8, 7.4) *p* = 0.001) were all significantly associated with acute inflammation. Mesenteric fat echogenicity (674(8.67, 52404) *p* = 0.009), submucosal layer thickness (79.9(2.16, 2951) *p* = 0.02) and mucosal layer thickness (13.6(1.54, 121) *p* = 0.02) were significantly associated with chronic inflammation. Submucosal layer echogenicity (*p* = 0.03), clarity (25.0(1.76, 356) *p* = 0.02) and mucosal layer thickness (53.8(3.19, 908) *p* = 0.006) were significantly associated with fibrosis. In multivariate analyses, wall and mucosal thickness remained significantly associated with acute inflammation (*p* = 0.02), mesenteric fat echogenicity with chronic inflammation (*p* = 0.009) and mucosal thickness (*p* = 0.006) with fibrosis.

**Conclusion:**

Multiple sonographic parameters are associated with histological phenotypes in Crohn’s disease although there is overlap between ultrasonic stigmata of acute inflammation, chronic inflammation and fibrosis.

**Graphic Abstract:**

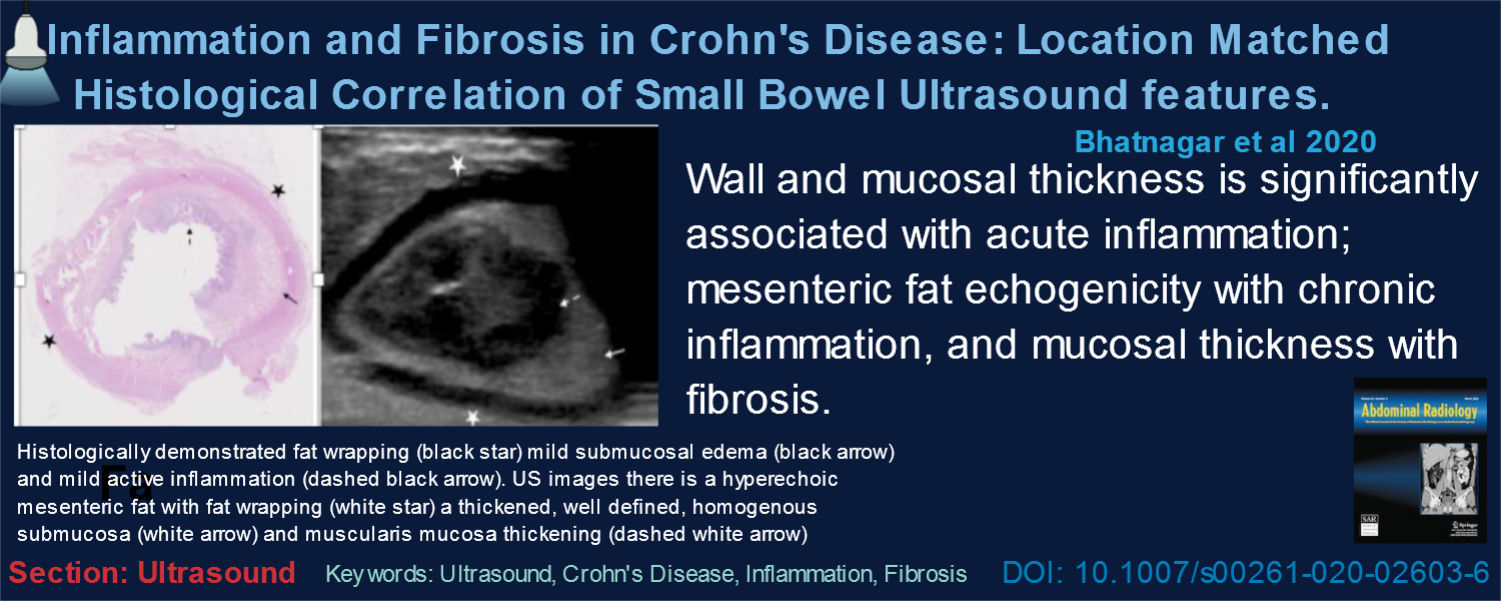

**Electronic supplementary material:**

The online version of this article (10.1007/s00261-020-02603-6) contains supplementary material, which is available to authorized users.

## Introduction

Cross-sectional imaging assessment of disease activity is fundamental to management of Crohn’s disease (CD) [1, 2]. Both small bowel ultrasound (SBUS) and magnetic resonance enterography (MRE) are accurate for detecting active disease [3–5] although SBUS, however, has several advantages, including patient acceptability [6], relative simplicity and general availability.

Studies supporting SBUS’s ability to assess disease activity have employed a variety of clinical, serological and endoscopic comparators [7] as a single standard of reference is lacking. The effectiveness of clinical indices such as the Crohn’s Disease Activity Index (CDAI) is increasingly questioned [8, 9], while colonoscopy and mucosal biopsies only evaluate superficial disease [10, 11] in at best, a limited length of terminal ileum. Transmural inflammation characterises CD [12], so histological analysis of resected small bowel specimens arguably serves as the most complete reference standard to validate imaging biomarkers: it looks beyond the mucosa, and allows comprehensive assessment of all intestinal layers, including quantification of mural fibrosis. Although resection specimens have been employed in several studies investigating CT and MRE [13–17], relatively few SBUS studies have used such a standard of reference [18–22]. These have mainly focused on a limited number of mural observations, such as Doppler signal or tested pre-defined definitions of activity. None has systematically evaluated the full range mural and extra mural observations apparent on SBUS in CD. The purpose of our study was to evaluate the utility of multiple mural and extramural sonographic features as potential imaging biomarkers of inflammation and fibrosis against a whole-mount histological section reference standard.

## Materials and methods

The study had research ethics committee approval. All patients gave written informed consent. Patients with a known diagnosis of CD over the age of 16 undergoing small bowel surgical resection between the dates of 01/01/2014 and 01/02/2015 were eligible. Members of the local research team identified suitable patients from the operating lists of two colorectal surgeons with a special interest in Crohn’s disease. Patients were excluded if they were pregnant (*n* = 0). In total, 16 patients were eligible, of whom four declined participation. The patient’s sex, age, Montreal classification, and reason for surgery were recorded.

### SBUS

Consenting patients underwent SBUS within 2 weeks of their planned operation date. Patients in whom the surgery was subsequently delayed beyond 2 weeks remained eligible providing they did not undergo any treatment changes, and the surgery was performed within 8 weeks of SBUS. Patients fasted for 4 h prior to SBUS and did not receive any oral or intravenous contrast. Two radiologists (20 years and 3 year’s experience in small bowel imaging) performed all ultrasounds in consensus. Prior imaging and clinical notes were reviewed before scanning to ensure the bowel segment scheduled for resection was interrogated. SBUS was undertaken on a standard platform (ELogiq E9, GE Medical Systems Ltd, Buckinghamshire, UK) using both curvilinear (C2-9) and high-resolution (9L and ML6-15) probes, including colour Doppler imaging (typical flow 6–9 m/s). Detailed observations (described below, see Online Appendix 1) were made for between 4 and 8 cross-sections through the abnormal small bowel in each patient. Sections were chosen to, as far as possible, encompass all the different US observations present in the diseased bowel segment. For example, if the bowel contained two different submucosal echogenicity patterns, both were included in the selected sections. Additionally, sonographically normal bowel sections were selected towards the periphery of diseased segment for comparison with diseased bowel.

**Fig. 1:**
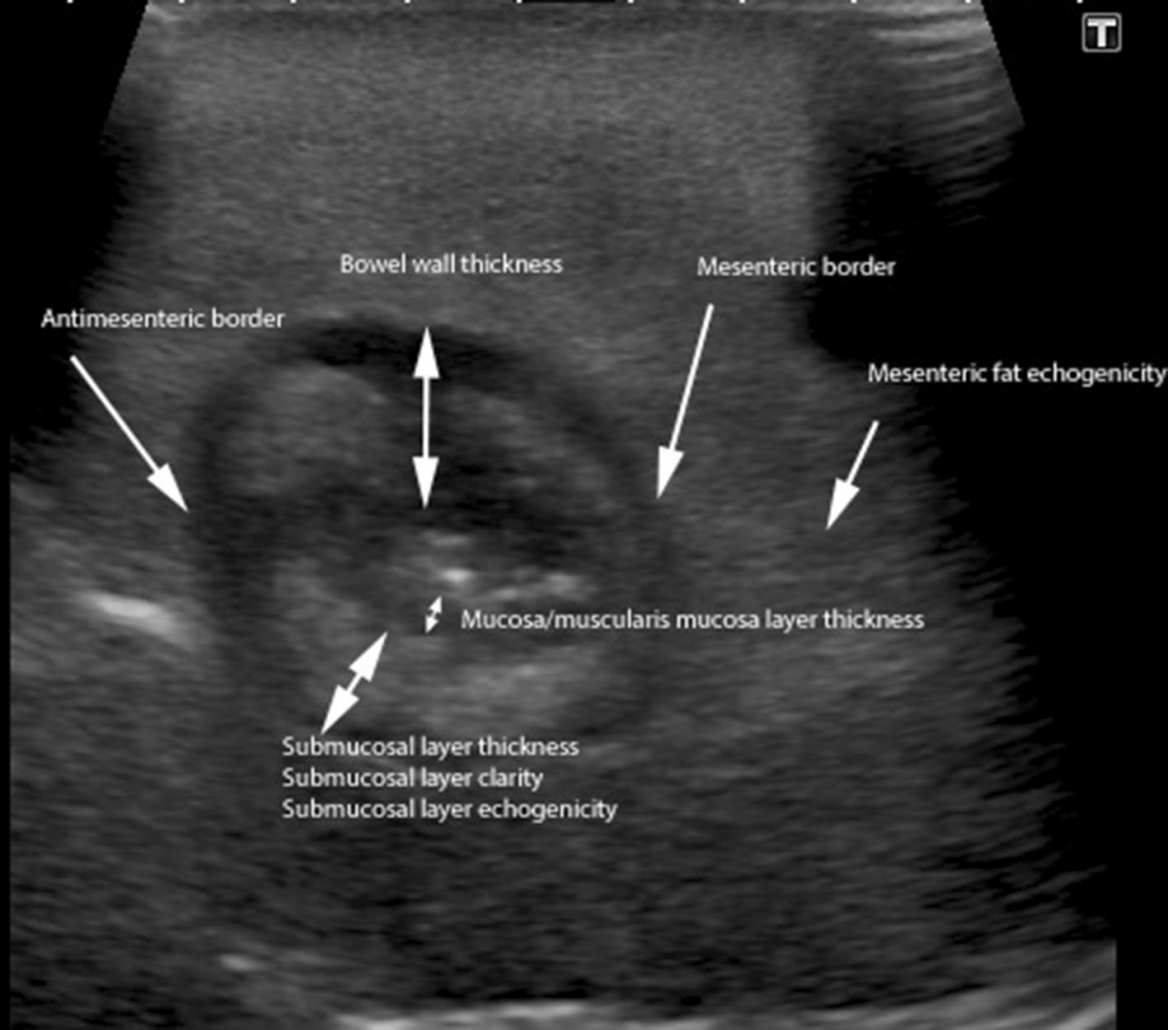
B-Mode US demonstrating multiple variables evaluated. Additionally, the presence of ulceration, increased Doppler vascular pattern and stenosis causing functional obstruction were recorded

Each SBUS section was systematically scored for multiple sonographic variables (for example wall thickness, mesenteric fat echogenicity, anti-mesenteric border clarity, submucosal layer echogenicity and mural Doppler vascular pattern) as detailed in Online Appendix 1 (Fig. 1). The observations were based on those already described in the literature, and the researchers own clinical experience. A pictorial and descriptive key was available to the radiologists for reference during their evaluation (Online Appendix 2).

### Co-localisation between US and histological sampling

The location of each chosen cross-section of the bowel was recorded by the interrogating radiologists with reference to distances from fixed structures such as Ileo-caecal valve or fistula (if present), using the method described by Punwani et al. [14]. To further facilitate co-localisation between bowel sections scored on SBUS and subsequent histological sectioning, the fixed resection specimen (and associated mesentery) was scanned by the same two radiologists who had performed the in vivo scan. Guided by the recorded locations, and considering any identifying sonographic characteristics of the bowel and/or surrounding tissues, the axial sections scored on the in vivo US were located on the surgical specimen and marked using blunt needles for subsequent histological sampling [14].

### Histological analysis

Histological assessment was performed by a specialist GI histopathologist (15 years of experience), blinded to the SBUS scoring and clinical information pertaining to underlying disease activity. Whole mount block sections taken through each identified cross-section were analysed after conventional H&E staining. For all histological analysis, abnormal bowel was compared to macroscopically “normal bowel” at the resection margins, which acted as an internal control. A previously described scoring system was used to classify the disease within the specimen. The inflammation was reported in each slice according to the method of Borley et al. as detailed in Online Appendix 3 [23]. Specifically, a histological acute inflammation score (AIS) was created by adding the scores for eosinophils, neutrophils, cryptitis, crypt destruction and ulceration. A histological chronic inflammatory score (CIS) was created by adding the scores for structural architectural changes, chronic inflammatory infiltrate, presence of granulomas and eosinophils in the lamina propria. These have been previously described in studies relating to Ulcerative colitis but applied to histological correlation studies in CD also [14, 24, 25].

Histological grading of mural fibrosis was undertaken on a three point scale, as previously described [13, 14] and detailed in Online Appendix 4. The specialist GI histopathologist further classified threshold values of the AIS (> 6), CIS (> 3) and fibrosis (> 0) for descriptive purposes only.

Of note, previous work has confirmed that mural thickness in formalin-fixed bowel specimens does not appreciably differ from that of fresh unfixed specimens, allowing direct comparisons of measured mural thickness between ultrasound and histology [14].

### Statistical analysis

All analyses examined the association between the SBUS parameters and the histological outcomes.

A feature of the data was that there were multiple measurements from each patient. This violated an assumption of independence, assumed by many statistical methods. To allow for these repeat measurements, the analysis was performed using multilevel regression methods.

AIS was considered a continuous outcome and analysed using multilevel linear regression. For this variable the results are presented as a regression co-efficient with 95% confidence intervals (Coefficient (95% CI)). The remaining outcomes (CIS and fibrosis score) were ordinal in nature and were analysed using multilevel ordered logistic regression (using the meologit command in the software program Stata 14, StataCorp LLC, Texas, USA). For these variables the results are presented as Odds Ratio with 95% confidence intervals.

The analyses were performed in two stages. First, the association between each separate SBUS parameter and each histological outcome was examined in a series of univariable analyses. Subsequently, the joint association of the parameters was examined in a multivariable analysis. Due to the relatively small sample size, the multivariable analysis was restricted only to those parameters showing some association with the outcome from the univariable analyses (*p* ≤ 0.1). A backwards selection procedure was used to retain only the statistically significant variables in the final model.

One patient scored category 4 (uniform hypoechoic mesenteric fat) for mesenteric fat echogenicity which was omitted from the results as the finding occurred only once and this was felt too infrequent to be reliably considered as a discrete category in itself. Agreement between histological and ultrasonic bowel wall thickness measurements was tested using Bland–Altman limits of agreement.

## Results

### Patient characteristics

Twelve patients (7 male, mean age 35) were included. Patients had a range of phenotypes according to the Montreal classification (Table 1). The mean time interval between the ultrasound and surgery was 9 days. All patients had been clinically stable in the pre-operative period with no alteration in medication and were not on corticosteroids.

**Table 1:** Patient characteristics, interval between ultrasound and surgery and reason for surgery

Patient	Age (years)	Sex	Montreal classification	Ultrasound scan to surgery (days)	Reason for surgery
1	27	M	A1L1B2	5	Obstructive stricture
2	37	F	A2L1B2	7	Obstructive stricture
3	35	M	A2L1B3	1	Abscess
4	24	M	A1L3B3p	1	Obstructive stricture
5	25	M	A1L1B2	5	Obstructive stricture
6	26	M	A1L3B3p	0	Failed medical management
7	45	F	A2L3B3	5	Obstructive stricture
8	69	M	A2L3B3	6	Obstructive stricture
9	34	F	A2L2B1	11	Failed medical management
10	23	F	A1L3B3	44	Fistula
11	38	M	A1L3B1	21	Failed medical management
12	36	F	A1L1B3	0	Obstructive stricture/adhesions
Mean	35			9	

### Specimen characteristics

In total, 50 small bowel cross-sections were scored from the 12 patients. Three to six sections were taken from individual resected segments with a median of 4 sections per segment (Online Appendix 5). The majority of sections demonstrated a combination of acute and chronic inflammation (AIS mean 7.6, range 0-14, CIS Mean 3.8, Range 0-6) and fibrosis (fibrosis score mean 0.6, range 0–2) (Online Appendix 5). Thirteen of the 50 sections demonstrated above threshold values for acute inflammation (> 6), chronic inflammation (> 3) and fibrosis (> 0), whilst a further 14 sections demonstrated above threshold values for both acute and chronic inflammation, but not fibrosis. Four sections demonstrated (above threshold values of) acute inflammation only, three sections demonstrated (above threshold values of) chronic inflammation only and six sections demonstrated (above threshold values of) fibrosis only.

#### Mural thickness

**Fig. 2:**
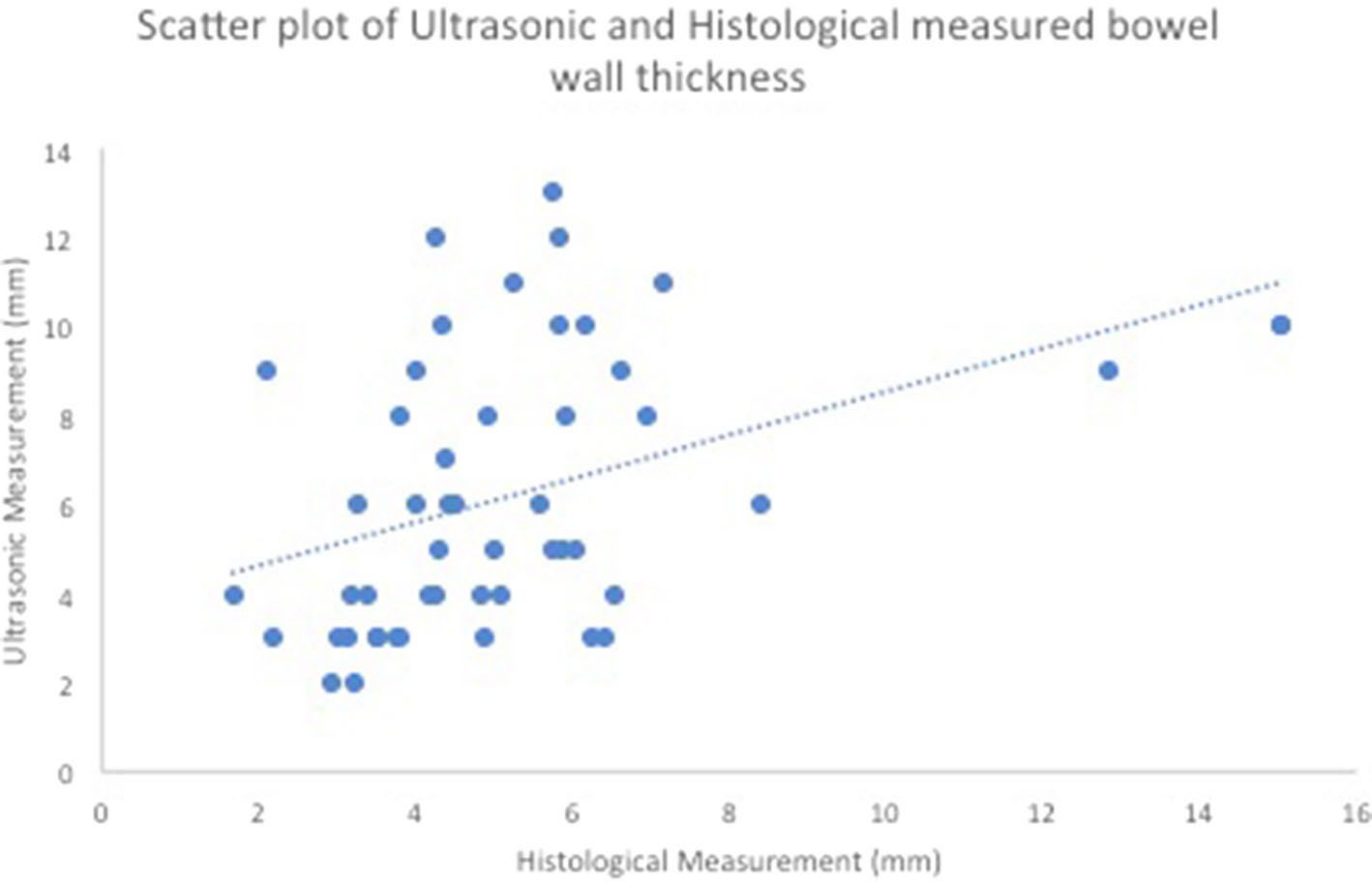
A scatter plot of ultrasonic against histological bowel wall thickness measurement

Overall, in vivo ultrasound estimates of wall thickness ranged from 3 to 12 mm (mean, 6.2 mm, median 5.5 mm) and histologically measured specimen wall thicknesses which ranged from 1.7 mm to 15 mm (mean 5.3 mm, median 4.7 mm). A scatter plot of ultrasonic against histological bowel wall thickness measurement is given in Fig. 2. The mean difference in measurements was 1 mm (95% Bland–Altman limits of agreement − 5 to 7 mm).

### Sonographic parameter correlation with histological acute inflammation

#### Univariate analysis

Bowel wall thickness, higher bowel wall thickness category, mesenteric fat echogenicity, submucosal layer thickness, submucosal layer clarity and mucosal layer thickness were all individually significantly associated with the AIS (Table 2).

**Table 2:** The association of sonographic variables with the acute inflammatory score (AIS) examined through univariable analyses

Variable	Category	Coefficient (95% CI)	*p* value
Wall thickness	–	0.8 (0.3, 1.3)	**0.001**
Wall thickening	0	0	**<** **0.001**
	1	4.8 (1.8, 7.7)	
	2	5.3 (0.0, 10.5)	
	3	7.7 (4.0, 11.3)	
Mesenteric fat echogenicity*	0	0	**0.005**
	1	4.7 (− 1.3, 10.7)	
	2	8.7 (3.0, 14.5)	
	3	7.4 (− 1.3, 16.1)	
Anti-mesenteric border definition	0	0	0.11
	1	2.0 (− 0.5, 4.4)	
Mesenteric border definition	0	0	0.54
	1 or 2	− 1.0 (− 4.0, 2.0)	
Submucosal thickness	0	0	**0.02**
	1	7.4 (1.2, 13.5)	
Submucosal echogenicity	0	0	0.16
	1	4.5 (0.0, 8.9)	
	2	4.6 (− 2.8, 12.0)	
	3	6.3 (0.8, 11.9)	
Submucosal clarity	0	0	**0.02**
	1	4.4 (0.6, 8.2)	
Mucosal layer thickness	0	0	**0.001**
	1 and 2	4.6 (1.8, 7.4)	
Mural Doppler pattern	0	0	0.85
	1 and 2	0.3 (− 2.8, 3.4)	

BWT is a continuous measurement whilst other categorical data is largely nominal with the exception of bowel wall thickening categories (BWTC), which is ordinal. The figures are the regression coefficients, and corresponding confidence intervals. For the one continuous variable (BWT) the coefficients indicate the change in outcome for one-unit increase in that measure. For the categorical variables, the coefficients indicate the mean difference in outcome between each category and a baseline category. *p*-values indicating the significance of the results are also presented

*Omitting one patient with category 4

Greater bowel wall thickness (and higher categories of bowel wall thickness categories) were associated with higher acute inflammatory scores. A 1 mm increase in wall thickness was associated with a 0.8 unit increase in the AIS.

Mesenteric fat echogenicity pattern one (focal hyperechoic without fat wrap), two (focal hyperechoic with fat wrap) and three (stratified) were associated with higher AIS than category zero (normal). Of these, pattern two had the strongest association with AIS which was almost 9 units higher than sections with normal mesenteric fat (pattern zero).

A thickened submucosa (submucosal layer thickness pattern one), an ill-defined submucosa (submucosal layer clarity pattern one) and a thickened mucosa (mucosal layer thickness patterns one or two) were associated with higher AIS. Specimens with a thickened submucosa (submucosal layer thickness pattern one) had AIS that were over seven units higher that of specimens with a normal submucosal thickness (submucosal layer thickness pattern zero).

**Fig. 3:**
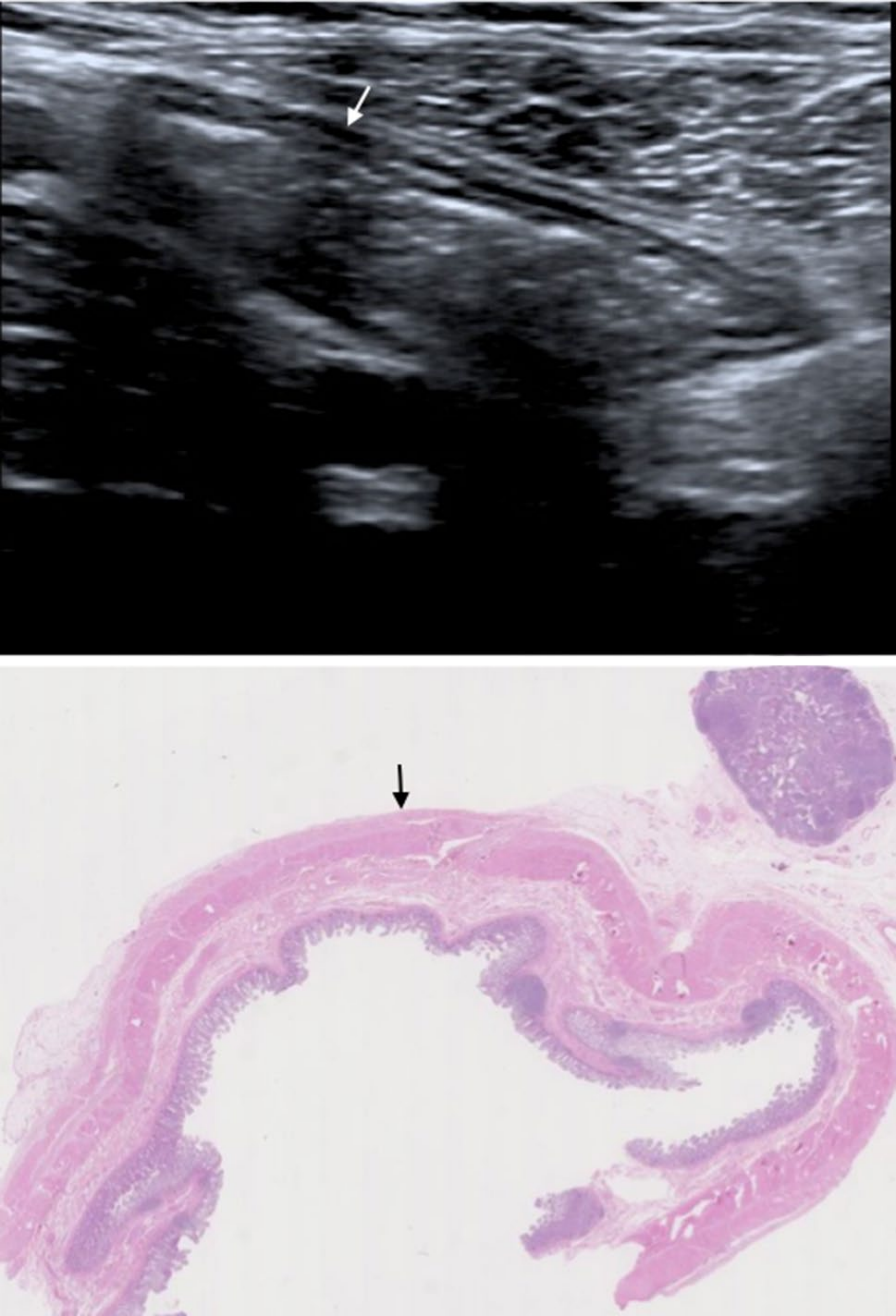
B-mode US image (TOP) with matched histological section. Normal mural thickness and mural echogenicity pattern (white arrow) on US. Histologically there are no significant levels of acute or chronic inflammatory markers or features of fibrosis (AIS 0, CIS 2 and fibrosis 0 (black arrow))

**Fig. 4:**
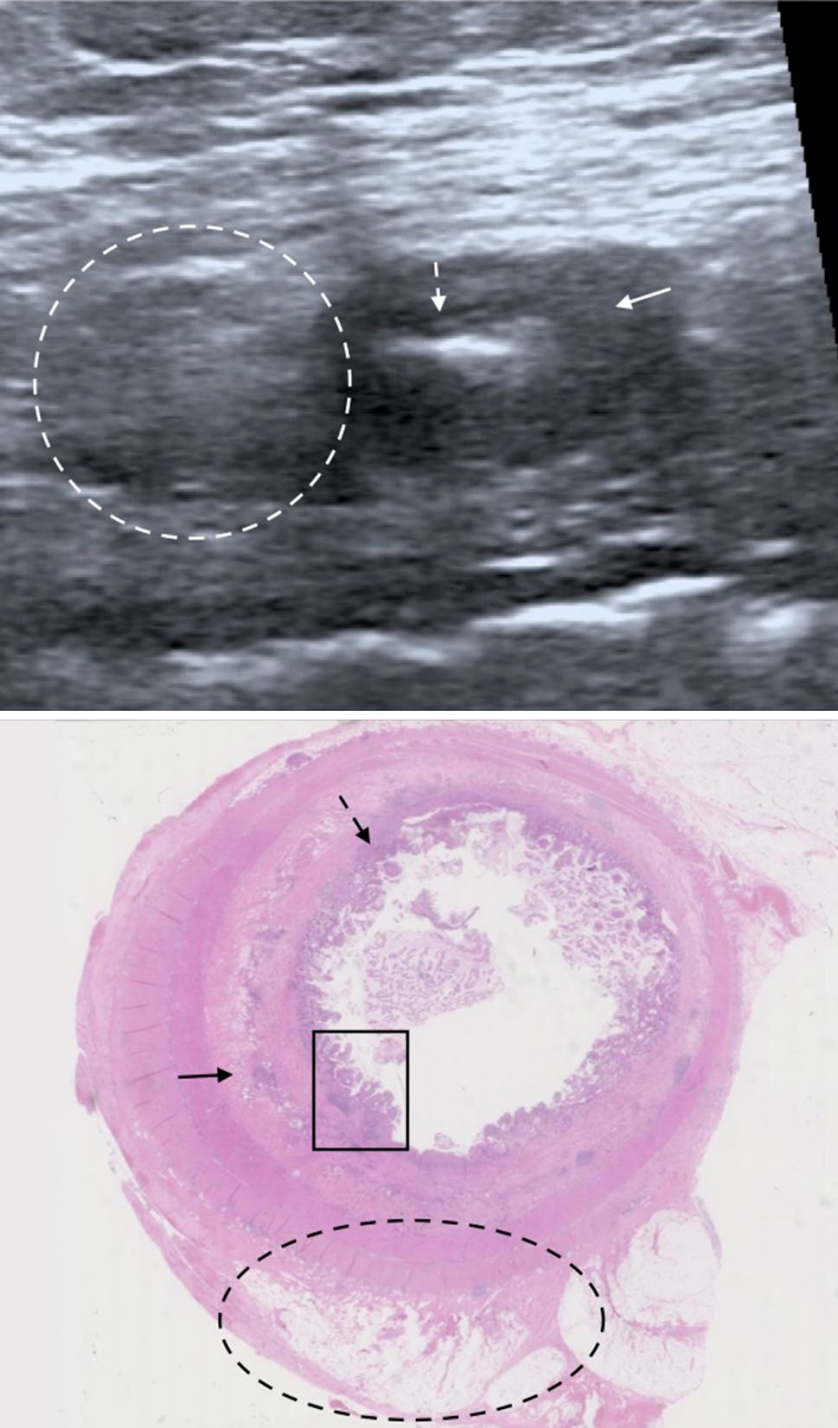
B-mode US image (TOP) with matched histological section. Acute, chronic and fibrotic sonographic and histopathological findings. Sonographically, there is hyperechoic mesenteric fat without fat wrapping (white dashed oval), a thickened, ill-defined submucosal layer of homogenously reduced echogenicity (solid white arrow) and a thickened mucosal layer (dashed white arrow). Histologically, there is mild congestion of the mesenteric fat (dashed black oval), the submucosa demonstrates oedema and inflammatory cell infiltrate (solid black arrow). There is a thickened muscularis mucosae (dashed black arrow). In the mucosa there is ulceration, crypt architectural changes and acute inflammatory infiltrate (black rectangle). AIS 14, CIS 4, fibrosis 1

**Fig. 5:**
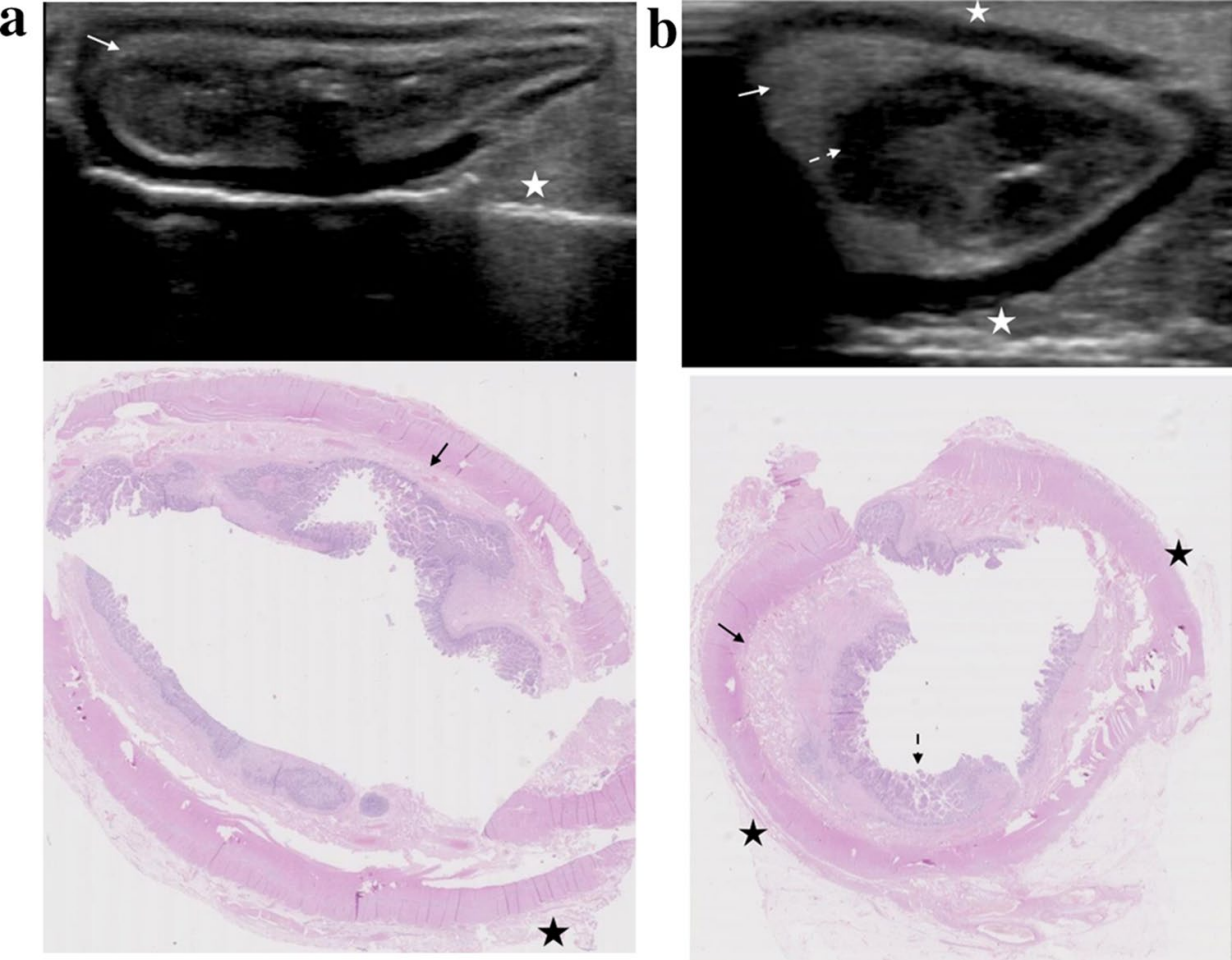
B-mode US images (TOP) with matched histological sections demonstrating the importance of fat wrapping and mucosal thickening in evaluating active inflammation. **a** A histologically normal segment (AIS 0, CIS 1, fibrosis 0) presents with hyperechoic mesenteric fat without fat wrapping (white star for US and black star for histology), a prominent, well defined, homogenous submucosa (white arrow for US and black arrow for histology) and a normal mucosa. **b** Histologically demonstrated fat wrapping (black stars showing extent), mild submucosal oedema (black arrow) and mild active mucosal inflammation (dashed black arrow) (AIS 8, CIS 4, fibrosis 1). Sonographically, there is hyperechoic mesenteric fat with fat wrapping (white stars showing extent), a thickened, well defined, homogenous submucosa (white arrow) and mucosal/muscularis mucosa thickening (dashed white arrow). Both images are acquired from the same patient

Images with matched sonographic and histological descriptors are provided as examples of normal (Fig. 3) and abnormal segments (Fig. 4, 5).

#### Multivariate analysis

The final multivariate model is summarised in Table 3.

**Table 3:** Multilevel regression model demonstrating the statistically significant sonographic variables associated with the histological acute inflammatory score (AIS)

Variable	Category	Coefficient (95% CI)	*p*-value
Wall thickness	–	0.6 (0.1, 1.1)	0.02
Mucosal layer thickness	0	0	0.02
	1 and 2	3.4 (0.6, 6.3)	

Both bowel wall thickness and mucosal layer thickness were found to be independently associated with the acute histological score. After adjusting for these two variables, no further variables were found to be statistically significant. As in the univariable analyses, a greater bowel wall thickness and a thickened mucosa with or without submucosal thickness (mucosal layer thickness patterns one or two) were associated with higher acute histological scores.

### Sonographic parameter correlation with histological chronic inflammation

#### Univariate analysis

Mesenteric fat echogenicity, submucosal layer thickness and mucosal layer thickness were statistically associated with the CIS (Table 4).

**Table 4:** The association of sonographic variables with the chronic inflammatory score (CIS) examined through univariable analyses

Variable	Category	Odds ratio (95% CI)	*p* value
Wall thickness	–	1.37 (0.94, 1.99)	0.10
Wall thickening	0	1	0.09
	1 or 2	9.45 (1.08, 82.9)	
	3	17.6 (1.10, 281)	
Mesenteric fat echogenicity*	0	1	**0.009**
	1	26.1 (0.40, 1688)	
	2	674 (8.67, 52,404)	
	3	131 (0.32, 54,209)	
Anti-mesentric border definition	0	1	0.24
	1	2.94 (0.50, 17.4)	
Mesentric border definition	0	1	0.82
	1 or 2	1.24 (0.20, 7.74)	
Submucosal thickness	0	1	**0.02**
	1	79.9 (2.16, 2951)	
Submucosal echogenicity	0	1	0.20
	1	32.1 (0.62, 1674)	
	2	199 (0.76, 51,659)	
	3	89.6 (1.13, 7116)	
Submucosal clarity	0	1	0.08
	1	14.5 (0.72, 295)	
Mucosal layer thickness	0	1	**0.02**
	1 and 2	13.6 (1.54, 121)	
Mural Doppler pattern	0	1	0.33
	1 and 2	2.87 (0.34, 24.2)	

The results are summarised in the form of odds ratios (and corresponding confidence intervals) due to the ordinal nature of CIS. The odds ratios represent the relative change in the odds of the next highest outcome score in one situation relative to another. For the one continuous variable (BWT) the coefficients indicate the change in the odds of being in the next category for one-unit increase in that measure. For the categorical variables, the odds ratios indicate the difference of being in the next outcome category for each ultrasound category relative to a baseline category. *p* values indicating the significance of the results are also presented

*Omitting one patient with category 4

All mesenteric fat patterns were associated with CIS, notably hyperechoic mesentery with fat wrapping (mesenteric fat echogenicity pattern two).

A thickened submucosa (submucosal layer thickness pattern one) and thickened mucosa with or without submucosal thickness (mucosal layer thickness patterns one or two) were associated with a higher CIS.

#### Multivariate analysis

Only mesenteric fat echogenicity was included in final model. No further variables were significant after factoring in this variable. As this was the only variable in the final model, the results are equivalent to those from the univariable analysis.

### Sonographic parameter correlation with histological fibrosis

#### Univariate analysis

Submucosal layer echogenicity, submucosal layer clarity and mucosal layer thickness were significantly associated with fibrosis (Table 5).

**Table 5:** The association of sonographic variables with the fibrosis score examined through univariable analyses

Variable	Category	Odds ratio (95% CI)	*p* value
Wall thickness	–	1.14 (0.83, 1.56)	0.44
Wall thickening	0	1	0.15
	1 or 2	25.0 (0.81, 770)	
	3	28.5 (0.57, 1419)	
Mesenteric fat echogenicity*	0	†	0.06
	1		
	2		
	3		
Anti-mesentric border definition	0	1	0.49
	1	0.56 (0.11, 2.91)	
Mesentric border definition	0	1	0.47
	1 or 2	0.44 (0.05, 4.13)	
Submucosal thickness	0	1	0.23
	1	5.93 (0.32, 110)	
Submucosal echogenicity	0	†	**0.03**
	1		
	2		
	3		
Submucosal clarity	0	1	**0.02**
	1	25.0 (1.76, 356)	
Mucosal layer thickness	0	1	**0.006**
	1 and 2	53.8 (3.19, 908)	
Mural Doppler pattern	0	1	0.50
	1 and 2	0.49 (0.06, 3.98)	

The results are summarised in the form of odds ratios (and corresponding confidence intervals) due to the ordinal nature of the fibrosis histological score. The odds rations represent the relative change in the odds of the next highest outcome score in one situation relative to another. For the one continuous variable (BWT) the coefficients indicate the change in the odds of being in the next category for one-unit increase in that measure. For the categorical variables, the odds ratios indicate the difference of being in the next outcome category for each ultrasound category relative to a baseline category. *p*-values indicating the significance of the results are also presented

*Omitting one patient with category 4

^†^Unable to calculate odds ratios as all results in one category having same outcome. Analysis using Kruskal–Wallis test

Submucosal layer echogenicity could not be analysed using regression methods as all sections with a normal submucosal echogenicity (submucosal layer echogenicity pattern zero) demonstrated no fibrosis. It was thus not possible to take account of the repeat measurements per patient and caution should be exercised when interpreting the significance of this association. The highest fibrosis scores were for sections with a reduced submucosal echogenicity (submucosal layer echogenicity pattern one) and for specimens with an increased submucosal echogenicity containing low echogenicity bands (submucosal layer echogenicity pattern three).

Sections with an ill-defined submucosa (submucosal layer clarity pattern one) had higher fibrosis scores than sections with a normal, well defined submucosa (submucosal layer clarity pattern zero). Additionally, specimens with a thickened mucosa with or without submucosal thickness (mucosal layer thickness patter one or two) had higher fibrosis scores than specimens with a normal mucosal thickness (mucosal layer thickness zero). A mucosal layer thickness pattern one or two was associated with 50 times greater odds of being in the next highest fibrosis category.

#### Multivariate analysis

Only mucosal layer thickness was included in final model. There was no additional effect of any other factors after adjusting for this variable. As this was the only variable in the final model, the results were equivalent to those from the univariable analysis.

## Discussion

We demonstrate that several sonographic features correlate with histologically graded acute inflammation, chronic inflammation and fibrosis. There is relatively little comparative data using full thickness pathological specimens in the literature. Previous comparisons have investigated Doppler ultrasound abnormalities only [18, 19], or intravenous contrast enhanced ultrasound (US) perfusion and bowel wall thickness [17]. Both Maconi et al. and Hata et al. pre-categorised sonographic findings of mural thickening and mural stratification patterns [20, 21], while Nylund et al. performed SBUS on the resected specimen only and reported their findings according to changes in the different mural layers [22].

We found that mucosal layer thickness was the only sonographic feature associated with all three histological categories. The mucosa is the primary layer involved in inflammation. Histologically there are a range of processes ranging from architectural distortion of the crypts at the mucosal surface, infiltration by inflammatory cells and mucin depletion [10, 12]. Mucosal healing is the goal of successful treatment [26], and our findings demonstrate the importance of evaluating the mucosa during sonography; the presence of a thickened mucosa suggests ongoing inflammation. Furthermore, studies have demonstrated hyperplasia of the muscularis mucosa in fibrosis which may in part explain why the mucosal layer is thickened in sections demonstrating fibrosis, with and without active inflammation [27]. Nylund et al. reported that it was possible to identify the muscularis mucosa (MM) on US in 48 of 69 sections where it was histologically thickened, and 52 of 54 sections where it was histologically normal. Their results suggested that submucosal fibrosis was more likely to be present with a non-visible (normal) MM than in those with a visible (thickened) MM [22].

Bowel wall thickness was significantly correlated with histological scores of acute inflammation, but interestingly not with chronic inflammation or fibrosis. Bowel wall thickness is the most frequently investigated parameter to assess disease presence and activity within the published literature [7]. Wilkens et al. have demonstrated that US is highly accurate in measuring bowel wall thickness, reporting that US measured mural thickness is on average 0.4 mm thicker than histological measurements, whilst MRE measurements are 1.4 mm thicker [17]. We also found reasonable agreement between ultrasound and histological bowel wall thickness measurement, albeit with some outliers. Previous studies across a range of imaging modalities have demonstrated bowel wall thickness is more strongly associated with active inflammation than fibrosis [13, 14]. For example, Moreno et al. compared sonographic findings with endoscopy and endoscopic biopsies in 178 segments (across 30 patients). Bowel wall thickness was the strongest US predictor of endoscopic remission with a normal bowel wall thickness (< 3 mm) predicting endoscopic remission with a PPV of 97.1% (77.1–99.7%) [28]. These findings are unsurprising as acute inflammation is associated with histological findings of oedema and inflammatory infiltrate which subsequently results in mural thickening [23]. However, studies using endoscopic biopsies cannot assess for any relationship between wall thickness and fibrosis, which requires full thickness specimen histology. Indeed, using histological specimens, Wilkens et al reported a significant correlation of 0.4 between ultrasonic bowel wall thickness measurements and histological fibrosis, although they found no such relationship using MRI measured wall thickness. Other MRE studies using full thickness specimen histology have however demonstrated a correlation between bowel wall thickness and fibrosis [15, 29], which is at odds with our findings. The dominant process in fibrosis is smooth muscle hyperplasia (in addition to proliferation of fibroblasts and myofibroblasts) which may not lead to the magnitude of mural thickening seen with acute inflammation [27]. However, it is reasonably established that fibrosis thickens the bowel wall so our negative findings are most likely due in part to the relatively small number of sections with fibrosis and the relatively limited histological scoring system used.

Abnormal patterns of mesenteric fat were associated with acute and chronic inflammation but not fibrosis. Our categorisation of mesenteric fat echogenicity incorporated various parameters including hypertrophy and fat wrapping. Mesenteric fat changes have infrequently been investigated in other histological correlation studies. Maconi et al. reported that patients with mesenteric fat hypertrophy exhibited higher clinical (CDAI) and serological (CRP and ESR) markers of inflammation than those without mesenteric fat hypertrophy [30]. This would support our findings that mesenteric fat abnormalities are linked to mural inflammation rather than mural fibrosis. Pallotta demonstrated that mesenteric fat hypertrophy was associated with the presence of fistulae at histology but not with the presence of strictures [31]. Seven of our cohort had obstructive strictures which histologically often exhibited a mixture of acute and chronic and inflammation, and only 1 had a fistulae. Our data therefore suggests stricturing disease is associated with mesenteric fat changes. Indeed, histologically, both fat wrapping and mesenteric thickening are known to be associated with acute and chronic inflammation [23]. It has been proposed that these processes may be a combination of at least two events: perivascular inflammation with mesenteric fibrosis and muscularis propria contraction (noting that mural fibrosis and mesenteric fibrosis are different entities). As such alterations in mesenteric fat echogenicity, represent transmural extension of more advanced Crohn’s disease [32]. Whilst many of the patients in our cohort presented with obstructive strictures all but one of the resected segments demonstrated features of acute or chronic inflammation or both.

We found that many sonographic features of the submucosal layer are also associated with inflammation and fibrosis in the univariate analyses. We tested both submucosal layer thickness, echogenicity and clarity as individual features. This arguably adds to the complexity of scoring system. Clarity specifically looks at the interface between the submucosa and the other bowel wall layers (as opposed to echogenicity) and may reflect a different pathological process, for example transmural cellular infiltration. Indeed, the fact that submucosal echogenicity and clarity had differing associations with the various histological findings does suggest they are giving, in part, different information. A thickened or an ill-defined submucosal was associated with active inflammation, while a thickened submucosal was also associated with chronic inflammation. Fibrosis was associated with reduced submucosal echogenicity, increased submucosal echogenicity with hypoechoic bands and an ill-defined submucosa. Hata et al. combined many of the individual sonographic criteria we describe into discrete patterns, and reported that mural thickening without stratification was associated with severe inflammation whilst mural thickening with stratification was associated with moderate inflammation [21]. Maconi et al concluded that stenosis with stratified or mixed echo pattern shows a significantly greater mucosal and submucosal fibrosis in comparison to a hypoechoic echo pattern [20]. Nylund et al. performed in vitro US on 14 resected small bowel segments and classified the submucosa into three categories (Echo-rich, Sporadic echo poor elements and diffuse echo poor elements) [22]. This parameter is comparable to our variable for submucosal layer echogenicity. They demonstrated that increasing echo poor elements in the submucosa were linked to fibrosis. These findings are confirmed by our study; we demonstrated that highest fibrosis scores were for specimens with a reduced submucosal echogenicity (pattern one) and for specimens with an increased submucosal echogenicity containing low echogenicity bands (pattern three).

Interestingly, we found no correlation between Doppler US findings and inflammation, and in this regard is at odds which much of the current literature. For example, In a study of 10 patients, Sasaki et al., reported significantly greater inflammatory cell infiltration (*p* = 0.043) and mural vascularity (*p* = 0.047) in specimens with increased mural Doppler flow [18]. None of the cohort were taking steroids which could have potentially help explain our findings by reducing acute inflammation. However, by definition, a patient cohort destined for surgery is likely to have received long term anti-inflammatory medication and have less active disease than a cohort prior to initiation of immunosuppressive therapy for example. The more chronic nature of our cohort may in part explain the lack of action between histological activity and doppler signal.

This study had limitations. The number of patients we evaluated were relatively low but at par with other studies reporting imaging and histological correlation in CD. We had numerous data points from the same patients for which we adjusted our statistical methodology, although this was not always possible. There was some minor variation in the number of histological sections (3–6, median 4) taken from individual resected segments, in order to maximise the variety of sonographic features being assessed. Whilst there is potential for individual patients to dominate the population, these seems unlikely given the relatively even spread of sections across the cohort. One finding, mesenteric fat hypertrophy was scored in a solitary category, 4 on a single occasion only. This was omitted from the assessment of this variable as it could not be reliably handled in the statistical analysis and as such this is a limitation. This was a single centre-study undertaken by the same two sonographers and the same histopathologist. Whilst this has the advantage of standardising interpretation it impacts the generisability of our findings especially given that US is anecdotally more operator dependent than other imaging modalities. We used a consensus process for the ultrasonic observation and did not test inter-observer variation. However, detailed analysis of inter-observer agreement between multiple reader for the various observations and recently been presented [33]. Whilst we used a well described histological scoring system, it is accepted that to date this (nor any other proposed histologically activity score) has not been validated [34]. Our study is affected by selection bias (as by definition, a minority of patients with CD who are surgically managed).

In conclusion, multiple sonographic parameters are associated with acute inflammation, chronic inflammation and fibrosis. As expected there is overlap in US findings, akin to histology. Segments that demonstrate mucosal, submucosal and mural thickening (> 3 mm) together with abnormal mesentery and loss of definition of the submucosal layer likely have underlying acute inflammation. Fibrosis is more likely to be present in segments with mucosal thickening, and if the submucosa is hypoechoic (either diffuse or focally) or ill-defined.

## Electronic supplementary material

Below is the link to the electronic supplementary material.

Supplementary material 1 (DOCX 19 kb)

Supplementary material 2 (PDF 1032 kb)

## Abbreviations

CD: Crohn’s disease
SBUS: Small bowel ultrasound
MRE: Magnetic resonance enterography
CDAI: Crohn’s Disease Activity Index
AIS: Histological acute inflammation score
CIS: Histological chronic inflammation score
MM: Muscularis mucosa
