# Supplementary material for: Inflammation and fibrosis in Crohn’s disease: location-matched histological correlation of small bowel ultrasound features
Source: Abdom Radiol (NY). 2020 Jun 20;46(1):144–55. doi: 10.1007/s00261-020-02603-6 (PMC7864849; doi:10.1007/s00261-020-02603-6)
Supplement: Supplementary file 1 — Supplementary material 1 (DOCX 19 kb) [file 261_2020_2603_MOESM1_ESM.docx]

Appendix 1: Multiple sonographic criteria used for scoring diseased sections

- Bowel wall thickness: continuous measurement
- Bowel wall thickness categories: 0:0-3mm, 1: 3-6mm, 2: 6-9mm, 3: >9mm
- Mesenteric fat echogenicity: 0: Normal, 1: Focal hyperechoic without fat wrap, 2: Focal hyperechoic with fat wrap, 3: Stratified heterogeneous with fat expansion, 4: Uniform hypoechoic
- Anti-mesenteric border: 0: Well defined, 1: Ill-defined
- Mesenteric border: 0: Well defined, 1: generally ill defined, 2: focally ill defined
- Submucosal layer thickness: 0: Normal, 1: Thickened
- Submucosal layer echogenicity: 0: Normal, 1: Reduced, 2 Uniform increased, 3: Increased with bands
- Submucosal layer clarity: 0: Normal - well defined, 1: Ill defined
- Mucosal layer thickness: 0: Normal - well defined, 1: Isolated thickened, 2: Thickened also in presence of submucosal thickening
- Mural Doppler pattern: 0: Normal, 1=increased focal (increased doppler signal affecting less than half the circumference on an axial image through the bowel) and 2 =diffuse increased doppler signal affecting more than half the circumference on an axial image through the bowel

Appendix 2: Pictorial and descriptive key available to radiologists for reference (see attached PDF file)

Appendix 3: Multiple histological criteria used for scoring diseased sections

- Factors for acute inflammation:
  - Acute inflammatory infiltrate (Eosinophils): 0: No increase, 1: Mild but unequivocal increase, 2: Moderate increase, 3: severe increase
  - Acute inflammatory infiltrate (Neutrophils): 0: No increase, 1: Mild but unequivocal increase, 2: Moderate increase, 3: severe increase
  - Neutrophils in the epithelium: 0: None, 1: <5% of crypts, 2: 5-50% of crypts, 3: >50% of crypts
  - Crypt destruction: 0: None, 1: Probable (local excess of neutrophils in part of crypts, 2: Probable – marked attenuation, 3: Unequivocal crypt destruction
  - Ulceration: 0: No erosion, ulceration/granulation tissue, 1: Regenerative epithelium adjacent to inflammation, 2: Early erosion, 3: Unequivocal erosion, 4: Ulcer or granulation tissue
- Factors for chronic inflammation:
  - Structural Architectural changes (villous/Crypt architecture): 0: Normal, 1: Mild, 2: Mild/Moderate or Multifocal, 3: Severe diffuse or Multifocal
  - Chronic inflammatory infiltrate (Summised from assessing the following features: Population of inflammatory cells and cell density, Depth of lymphoid aggregates, wall thickness score and perineural inflammation score): No increase, 1: Mild but unequivocal increase, 2: Moderate increase, 3: severe increase
  - Eosinophils in the lamina propria: No increase, 1: Mild but unequivocal increase, 2: Moderate increase, 3: severe increase
  - Granulomas: Yes or No

Appendix 4: Histological grading criteria for fibrosis

- Grade 0: (none) absence of fibrosis or minimal fibrosis limited to the mucosa [<25%]
- Grade 1 (mild/moderate) Mild stricture with non-dilated lumen with submucosal fibrosis [>25%] and or muscular hyperplasia with preserved layers
- Grade 2 (severe) Massive transmural fibrosis and/or effacement of normal layers and/or severe stricture

Appendix 5: Table 6: Histological section characteristics. Acute inflammatory score (AIS) is summarised from acute inflammation, acute inflammation, cryptitis, crypt destruction and ulceration, Chronic inflammatory score (CIS) is summarised from chronic inflammation and architectural distortion.

| **Section** | **AIS** | **CIS** | **Fibrosis** |
| --- | --- | --- | --- |
| 1a | 1 | 3 | 2 |
| 1b | 0 | 3 | 2 |
| 1c | 0 | 3 | 1 |
| 1d | 0 | 3 | 0 |
| 2a | 1 | 4 | 0 |
| 2b | 7 | 4 | 0 |
| 2c | 11 | 4 | 0 |
| 2d | 12 | 4 | 0 |
| 2e | 13 | 5 | 0 |
| 3a | 10 | 4 | 1 |
| 3b | 13 | 5 | 0 |
| 3c | 13 | 5 | 0 |
| 3d | 9 | 5 | 0 |
| 4a | 12 | 5 | 1 |
| 4b | 12 | 6 | 1 |
| 4c | 12 | 6 | 0 |
| 4d | 14 | 6 | 0 |
| 5a | 3 | 3 | 1 |
| 5b | 11 | 5 | 2 |
| 5c | 0 | 3 | 1 |
| 5d | 0 | 3 | 0 |
| 6a | 12 | 6 | 0 |
| 6b | 12 | 6 | 0 |
| 6c | 12 | 6 | 0 |
| 6d | 12 | 6 | 0 |
| 7a | 5 | 4 | 1 |
| 7b | 8 | 4 | 1 |
| 7c | 0 | 1 | 0 |
| 7d | 0 | 1 | 0 |
| 8a | 8 | 3 | 0 |
| 8b | 11 | 6 | 1 |
| 8c | 12 | 6 | 1 |
| 8d | 12 | 6 | 0 |
| 8e | 11 | 6 | 1 |
| 8f | 14 | 6 | 2 |
| 9a | 3 | 4 | 0 |
| 9b | 0 | 2 | 0 |
| 9c | 0 | 2 | 0 |
| 10a | 14 | 6 | 1 |
| 10b | 12 | 3 | 1 |
| 10c | 12 | 3 | 2 |
| 10d | 7 | 0 | 2 |
| 11a | 14 | 4 | 1 |
| 11b | 13 | 4 | 1 |
| 11c | 14 | 4 | 1 |
| 12a | 0 | 0 | 0 |
| 12b | 5 | 2 | 1 |
| 12c | 4 | 2 | 1 |
| 12d | 0 | 0 | 0 |
| 12e | 0 | 0 | 0 |
