# Supplementary material for: Inflammation and fibrosis in Crohn’s disease: location-matched histological correlation of small bowel ultrasound features
Source: Abdom Radiol (NY). 2020 Jun 20;46(1):144–55. doi: 10.1007/s00261-020-02603-6 (PMC7864849; doi:10.1007/s00261-020-02603-6)

METRIC

Ultrasound Imaging Key

Scoring guide to be used in conjunction with ultrasound CRF pictorial definitions.

Bowel wall thickness	
Score	
0	0-3mm - Normal
1	3-6mm
2	6-9mm
3	>9mm

Functional obstruction = Upstream dilatation	
Score	
0	None
1	Present

Mesenteric fat echogenicity	
Score	
0	Normal
1	Focal hyperechoic without fat wrap (i.e. focal defined area of mesenteric fat of increased echogenicity without overall increase in volume of peri mural fat)
2	Focal hyperechoic with fat wrap (i.e. focal defined area of mesenteric fat of increased echogenicity with overall increase in volume of peri mural fat)
3	Stratified heterogeneous with fat expansion (chronic disease) - overall increase in volume of peri mural fat with maintained normal mesenteric stratification and no focal hyperechoic area)
4	Uniform hypoechoic (i.e. mesenteric fat of decreased echogenicity)

Anti-mesenteric border	
Score	
0	Well defined (smooth interface between bowel wall and mesenteric fat)
1	Ill defined (irregular Interface between bowel wall and mesenteric fat)

Mesenteric border	
Score	
0	Well defined (smooth interface between bowel wall and mesenteric fat)
1	Generally ill defined (generally irregular Interface between bowel wall and mesenteric fat)
2	Focally ill defined (focal irregular Interface between bowel wall and mesenteric fat)

Submucosal layer thickness	
Score	
0	Normal
1	Thickened (increased thickness compared to submucosa of normal bowel in the same patient)

Submucosal layer echogenicity	
Score	
0	Normal
1	Reduced (echogenicity lower than normal bowel in same patient)
2	Uniform increased (echogenicity greater than normal bowel in same patient)
3	Increased with bands (echogenicity greater than normal bowel in same patient with spoke wheel hypoechoic bands)

METRIC

Submucosal layer clarity	
Score	
0	Normal - well defined (smooth interface between submucosa and other wall layers)
1	Ill defined (irregular interface between submucosa and other wall layers)

Mucosal layer	
Score	
0	Normal - well defined
1	Isolated thickened (increased thickness compared to mucosa of normal bowel in the same patient)
2	Thickened also in presence of submucosal thickening

Ulceration	
Score	
0	None
1	Superficial-< 50% wall thickness
2	Deep-≥50% wall thickness

METRIC

Doppler vascular pattern imaged in axial section	
Score	
0	Normal
1	Increased focal (increased Doppler signal isolated to less than half the circumference on a trans-axial image) compared to normal bowel in the same patient)
2	Increased generalised (increased Doppler signal affecting more half the circumference on a trans-axial image compared to normal bowel in the same patient)

Peristalsis related to stricturing	
Score	
0	Distension of the segment during peristaltic wave
1	Non distension of segment during proximal peristaltic wave

Disease activity
At least one of: wall thickening/ focal hyperechoic mesentery (with or without fat wrap)/ isolated mucosal thickening /ill defined submucosal layer/ ill defined anti-mesenteric border/ Increased Doppler vascular pattern/ulceration/abscess

Segmental disease severity assessment	
Score	
0	None
1	Early-superficial ulceration and/or mild wall thickening/ mild increased vascularity
2	Advanced-transmural disease and/or fistulation and/or stricturing and/or cobblestoning/ and or wall oedema or significant thickening

Lymphadenopathy (all measurements in short axis)	
Score	
0	None
1	Cluster all less than 1cm
2	1 or 2 nodes > 1cm
3	3 or more nodes greater than 1cm

METRIC

Ultrasound Imaging Key

Mesenteric Fat Echogenicity

0 - Normal

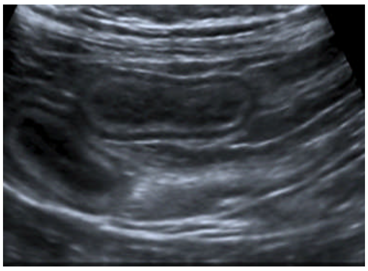

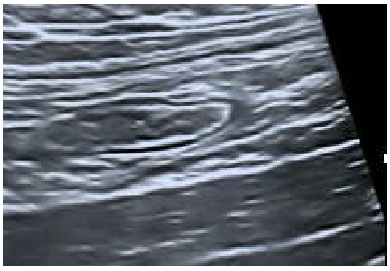

1 - Focal hyperechoic without fat wrap (i.e. focal defined area of mesenteric fat of increased echogenicity without overall increase in volume of peri mural fat)

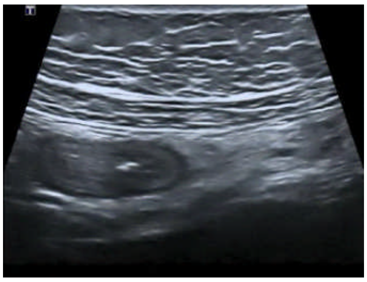

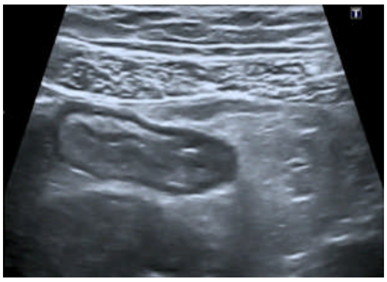

2 - Focal hyperechoic with fat wrap (i.e. focal defined area of mesenteric fat of increased echogenicity with overall increase in volume of peri mural fat)

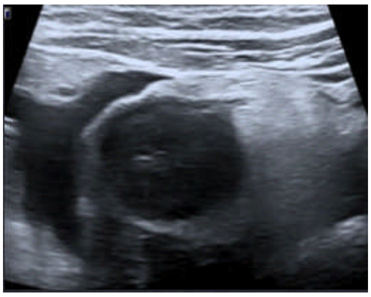

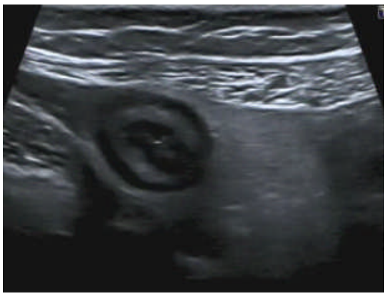

3 - Stratified heterogeneous with fat expansion (chronic disease) - overall increase in volume of peri mural fat with maintained normal mesenteric stratification and no focal hyperechoic area)

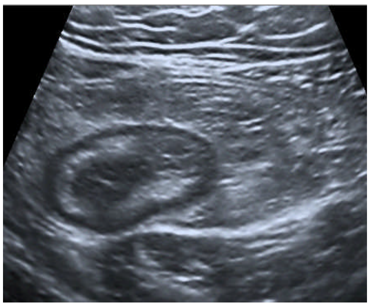

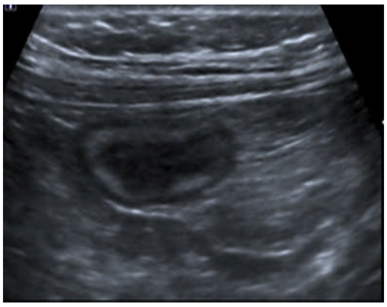

4 - Uniform hypoechoic (i.e. mesenteric fat of decreased echogenicity)

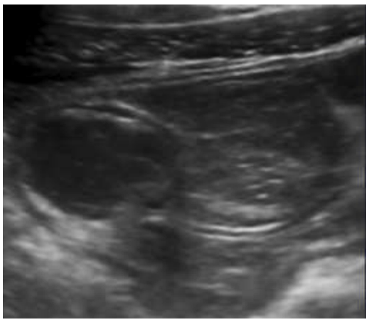

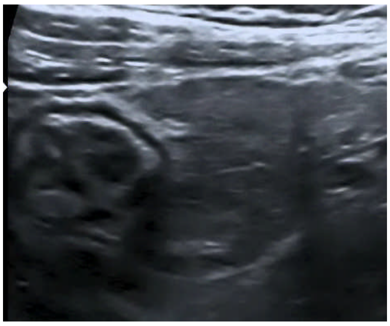

METRIC

Ultrasound Imaging Key

Anti-Mesenteric Border

Example 1

0 - Well defined (smooth interface between bowel wall and mesenteric fat)

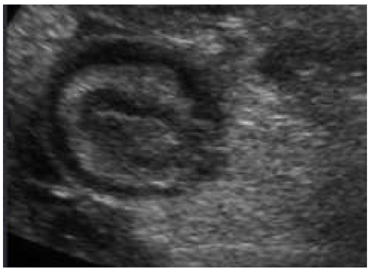

Post treatment

1 - Ill defined (irregular Interface between bowel wall and mesenteric fat)

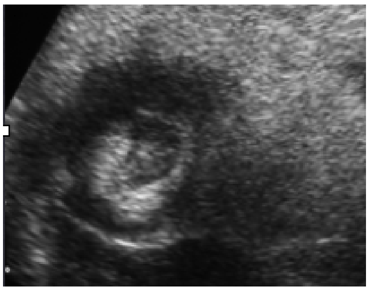

Pre treatment

Example 2

0 - Well defined (smooth interface between bowel wall and mesenteric fat)

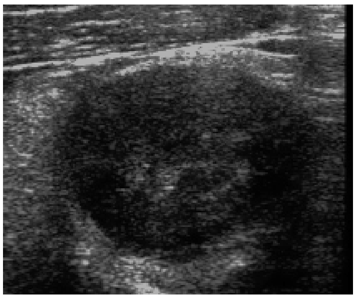

Post treatment

1 – Ill defined (irregular Interface between bowel wall and mesenteric fat

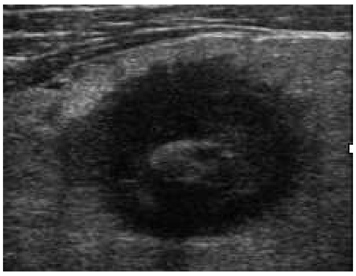

Pre treatment

METRIC

Ultrasound Imaging Key

Mesenteric Border

0 - Well defined

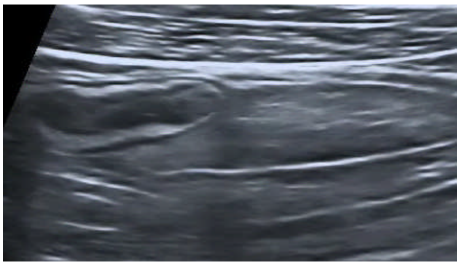

1 - Ill defined

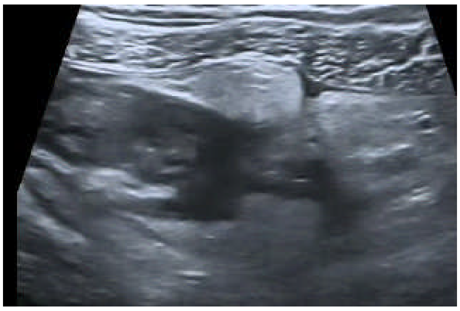

2 - Focal involvement

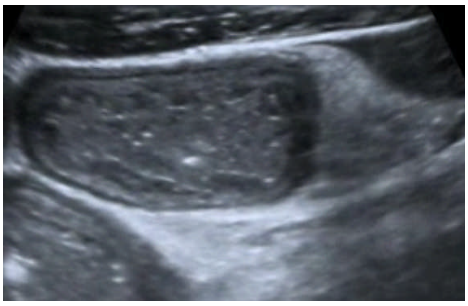

Submucosal layer thickness

Ileostomy

0 - Normal

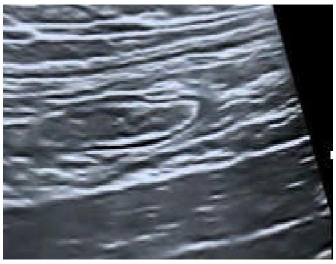

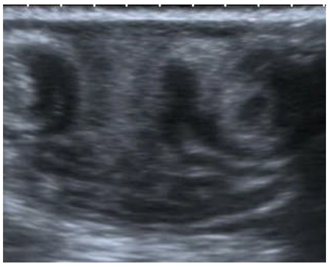

1 - Thickened (increased thickness compared to submucosa of normal bowel in the same patient

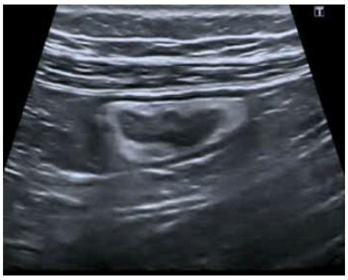

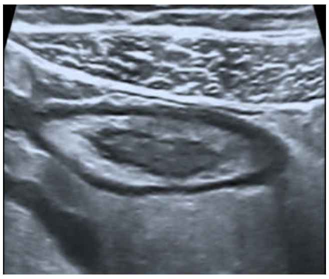

METRIC

Ultrasound Imaging Key

Sub-mucosal layer echogenicity

0 - Normal

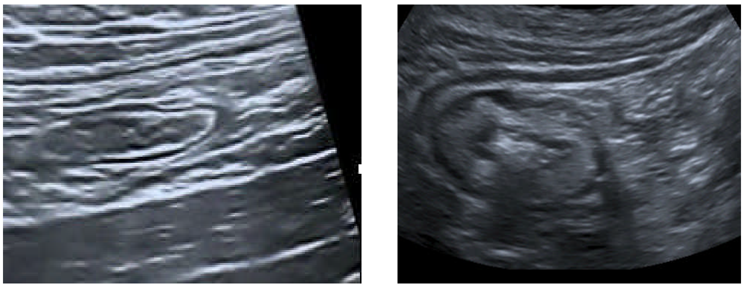

1 - Reduced (echogenicity lower than normal bowel in same patient)

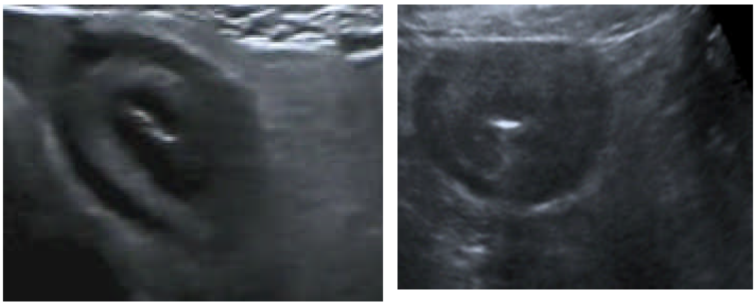

2 - Uniform increased (echogenicity greater than normal bowel in same patient)

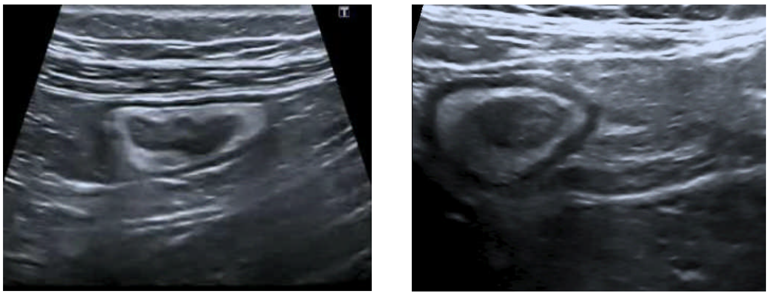

3 - Increased with bands (echogenicity greater than normal bowel in same patient with spoke wheel hypoechoic bands)

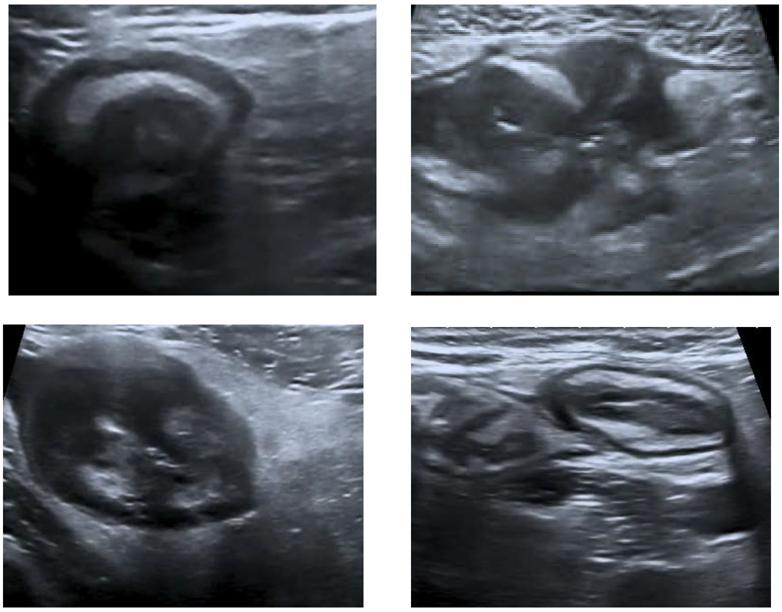

METRIC

Ultrasound Imaging Key

Sub-mucosal Layer Clarity

0 - Normal well defined (smooth interface between submucosa and other wall layers)

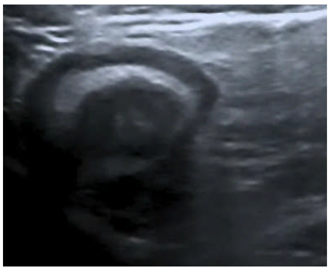

1 - Ill defined (irregular interface between submucosa and other wall layers)

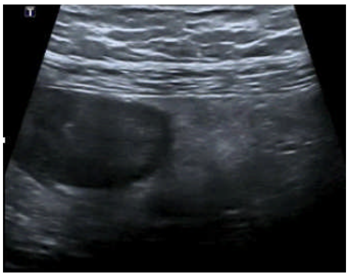

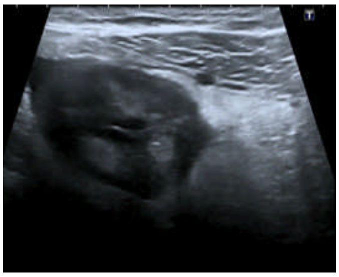

Mucosal layer

0 - Normal, well defined

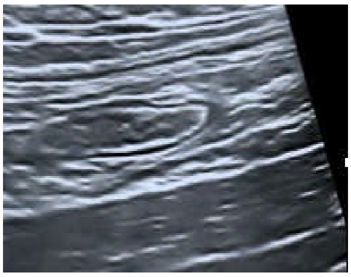

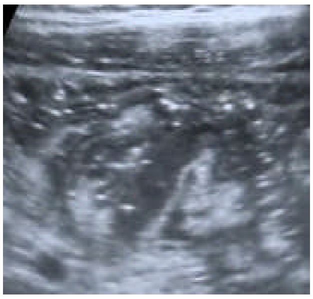

ileocaecal valve ulceration

1 - Isolated thickened (increased thickness compared to mucosa of normal bowel in the same patient

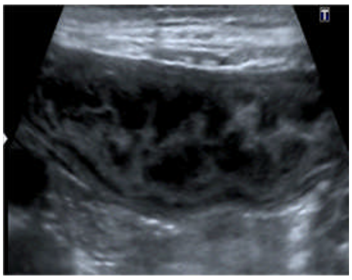

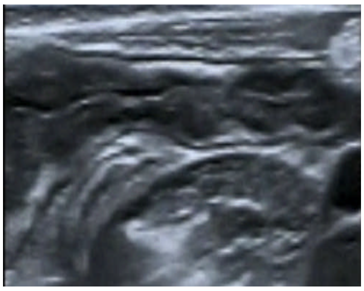

2 - Thickened also in the presence of submucosal thickening

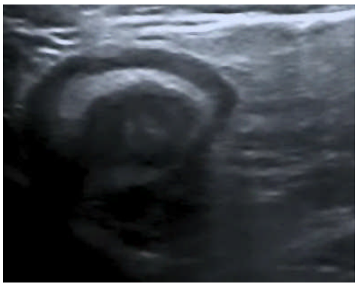

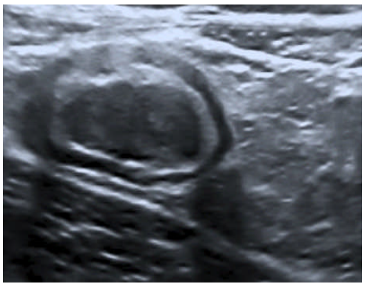

METRIC

Ultrasound Imaging Key

Doppler Vascular Pattern Imaging In Axial Section

0 - Normal

1 - Increased focal (increased Doppler signal isolated to less than half the circumference on a trans-axial image) compared to normal bowel in the same patient)

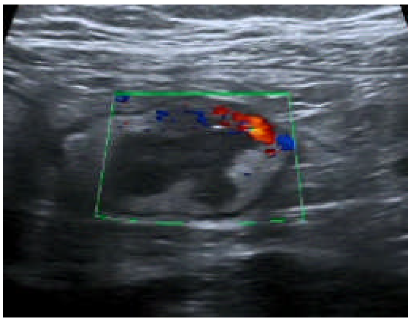

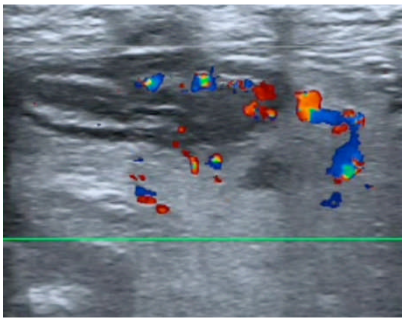

2 - Increased generalised (increased Doppler signal affecting more half the circumference on a trans-axial image compared to normal bowel in the same patient)

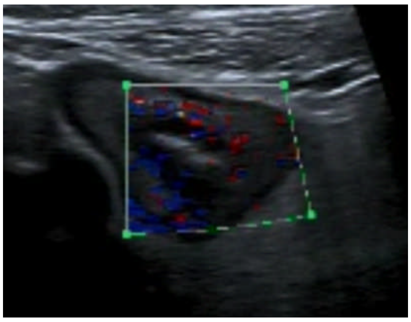

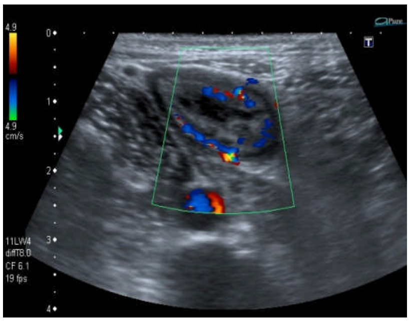

Ulceration

0-Normal

1-Superficial-< 50% wall thickness

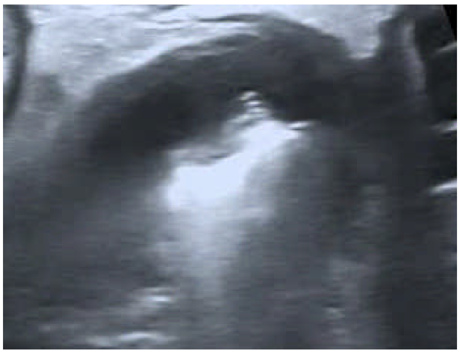

2-Deep-≥50% wall thickness

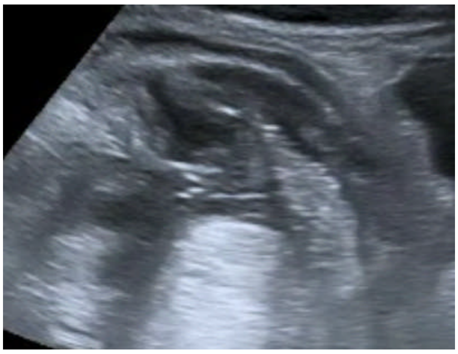

METRIC

Ultrasound Imaging Key

Functional obstruction = upstream dilatation

Lymphadenopathy

0=none. 1=cluster all less than 1cm. 2=cluster 1 or2 nodes >1cm. 3=3 or more nodes greater than 1cm

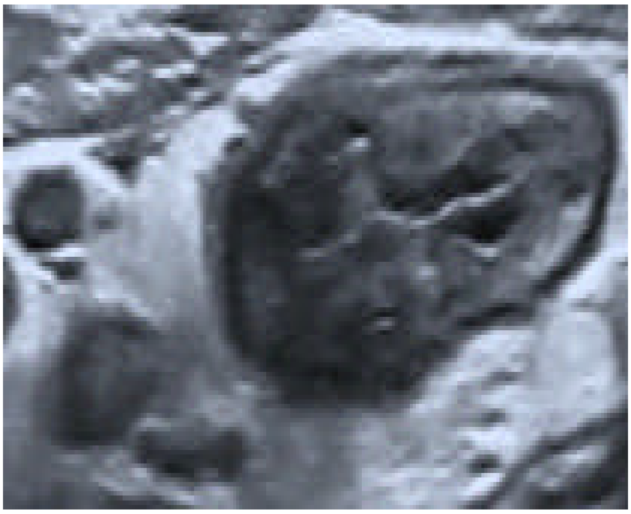

Supplement: Supplementary file 2 — Supplementary material 2 (PDF 1032 kb) [file 261_2020_2603_MOESM2_ESM.pdf]
